# The Use of Spreadsheets for Pharmacokinetic Simulations

**DOI:** 10.1100/tsw.2003.02

**Published:** 2003-04-21

**Authors:** Joseph Chamberlain

**Affiliations:** 127 Church Green Road, Bletchley, Milton Keynes, Buckinghamshire, MK3 6DE, UK

**Keywords:** pharmacokinetics, simulations, spreadsheets, drug metabolism, multiple dosing, gastrointestinal absorption, drug distribution, enterohepatic recycling, bioavailability

## Abstract

The use of simple spreadsheets is described to create simulations of complex pharmacokinetic phenomena. The basics of spreadsheets are first described and are developed to demonstrate classical pharmacokinetics without the use of differential or integral calculus. Using standard spreadsheet commands, the technique is shown to be applicable to the full range of advanced pharmacokinetic simulations. Demonstrations of the effect of a variety of physiological eventualities are included to show the versatility of the technique. The technique is very simple to use and is always in the complete control of the modeller.

### INTRODUCTION

Knowledge of pharmacokinetic principles and physiological processes enables research workers to simulate the fate of drugs in humans or animals by the use of appropriate pharmacokinetic equations. However, even a relatively simple pharmacokinetic model (single absorption site, central compartment, distributed compartment, eliminated compartment) leads to complex mathematical equations. Commercial packages are available to make this process easier, but such packages may be expensive or relatively inflexible. This paper describes the use of standard spreadsheet programs to simulate complex pharmacokinetics while still leaving the experimenter in complete control of the model. The paper describes simple classical pharmacokinetics, including multiple dosing and infusions, and describes how the model can be developed for an array of physiological eventualities.

### MATERIALS AND METHODS

The reader needs only a standard spreadsheet package (Microsoft EXCEL is used here but almost any commercial spreadsheet can be use), an understanding of basic pharmacokinetics, and a desire to understand more of the physiological processes involved in the fate of drug molecules in the organism.

### DEVELOPMENT OF THE METHOD

#### Simple Spreadsheet Principles

As a simple introduction to the use of spreadsheets in simulation, Table 1 illustrates the equations placed in the cells of a spreadsheet to simulate simple first-order decay: the type of curve expected for disappearance of drug from an absorption site or elimination from a central compartment.

**TABLE 1**
**Spreadsheet Formulae for Simulation of First-Order Decay**

| | A | B | C | D | E |
|---|---|---|---|---|---|
| **1** | Checksum | Time | Gut (absorption site) | Blood (central site) | $K_a$ (absorption coefficient) |
| **2** | =SUM(C2.D2) | 0 | 100 | 0 | 0.33 |
| **3** | =SUM(C3.D3) | =B2 + 1 | =C2 - C2*$E$2 | =D2 + C2*$E$2 | |
| **4** | =SUM(C4.D4) | =B3 + 1 | =C3 - C3*$E$2 | =D3 + C3*$E$2 | |

Experienced users of spreadsheets will recognise that line three is obtained simply by copying and pasting the relevant sections of line 2. The remainder of the spreadsheet is obtained by copying the same formulae into subsequent lines, as many as desired, depending on the length of the simulation. The following is a brief description of the formulae in each of these initial cells.

Cell E2 contains a value for the coefficient of absorption ($K_a$). In this case the value is set at 0.33, which means for each time segment of the simulation (arbitrary units), 33% of the total existing value at the absorption site is absorbed; this is an exaggerated value as only 10 time points are included in this demonstration..

Cell A2 gives the sum of the values in cells C2 to D2; the importance of including this calculation will become apparent when complex models are created.

Cell B2 contains the value 0 and represents the 0 time for the simulation. Cell B3 increases this value by unity to give the second time point, which for the purpose of this demonstration is in arbitrary units.

Cell C2 contains the value 100, the starting value for the simulation. Cell C3 gives the amount remaining after the first time interval.

**TABLE 2**
**Calculated Values after Running the Spreadsheet Shown in Table 1**

| Checksum | *Time* | Gut | Blood | Ka |
|---|---|---|---|---|
| 100 | 0 | 100 | 0 | 0.33 |
| 100 | 1 | 67 | 33 | |
| 100 | 2 | 44.89 | 55.11 | |
| 100 | 3 | 30.0763 | 69.9237 | |
| 100 | 4 | 20.15112 | 79.84888 | |
| 100 | 5 | 13.50125 | 86.49875 | |
| 100 | 6 | 9.045838 | 90.95416 | |
| 100 | 7 | 6.060712 | 93.93929 | |
| 100 | 8 | 4.060677 | 95.93932 | |
| 100 | 9 | 2.720653 | 97.27935 | |
| 100 | 10 | 1.822838 | 98.17716 | |

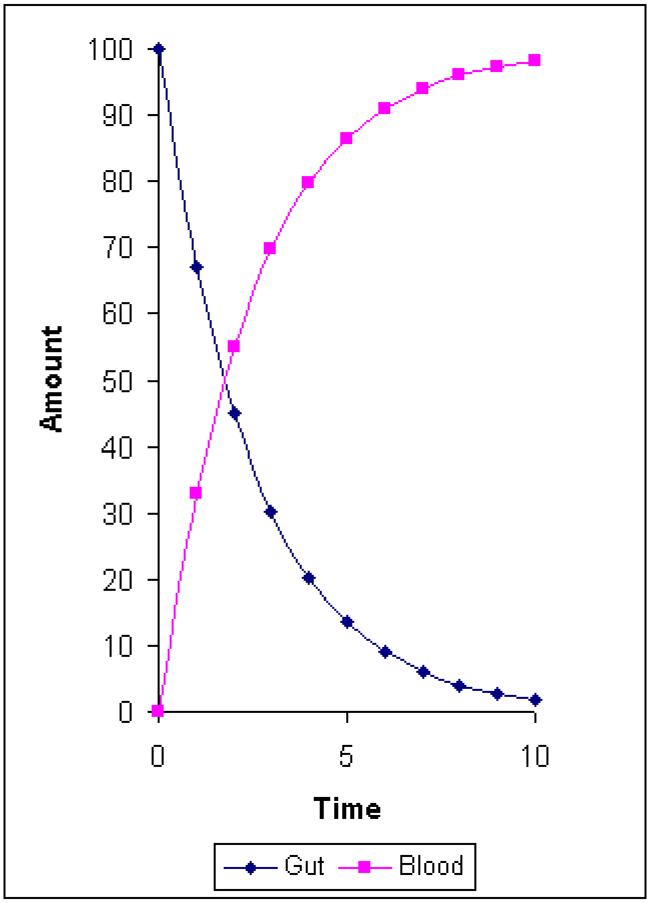

**FIGURE 1**. Typical first-order decay from plot of values in Table 2.

Cell D2 contains the value 0, the starting value for the central compartment that receives the absorbed molecules

Table 2 shows the outcome of running this spreadsheet for 10 time units, and Fig. 1 is a plot of the values showing a typical first-order decay.

Fig. 1 represents the absorption of drug from the gut to the blood by simple first-order kinetics. The value of the absorption coefficient ($K_a$) can be varied to show faster or slower absorption. The checksum column confirms the total values in the cells at a particular timepoint remains constant, i.e., the model is in balance.

### Absorption Followed by Elimination

The next step is to add an elimination process and the required equations are shown in Table 3. The resulting calculated values are shown in Table 4, where $K_a$ and $K_e$ are both equal to 0.5, and the corresponding plots are shown in Fig. 2.

It can now be seen that this very simple spreadsheet procedure can rapidly produce the classical pharmacokinetic profiles from $K_a$ and $K_e$ values. Although the same profiles can be obtained from conventional integration, any simulations of more complex models require equations of ever-increasing complexity. Using the methods described here, complex models can be simulated just by writing separate expressions for all the forces acing on a particular element of the model. The device to sum the values in all elements is a check that the model is in balance as the development progresses.

**TABLE 3**
**Spreadsheet Formulae for Simulation of Absorption and Elimination, Both with First-Order Kinetics**

| | A | B | C | D | E | F |
|---|---|---|---|---|---|---|
| **1** | Checksum | Time | Gut (absorption site) | Blood (central site) | Urine (drug eliminated) | $K_a$ (absorption coefficient) |
| **2** | =SUM(C2.E2) | 0 | 100 | 0 | 0 | 0.5 |
| **3** | =SUM(C3.E3) | =B2 + 1 | =C2 - C2*$F$2 | =D2 + C2*$F$2 - D2*$F$4 | =E2 + D2*$F$4 | $K_e$ (elimination coefficient) |
| **4** | =SUM(C4.E4) | =B3 + 1 | =C3 - C3*$F$2 | =D3 + C3*$F$2 - D3*$F$4 | =E3 + D3*$F$4 | 0.5 |

## ADVANCED PHARMACOKINETICS

We can now turn to slightly more complex models usually covered in 'advanced' pharmacokinetic treatises. All spreadsheets described below are included in the attached file SpiresAdam; illustrations here are taken from the file by varying the parameters appropriately.

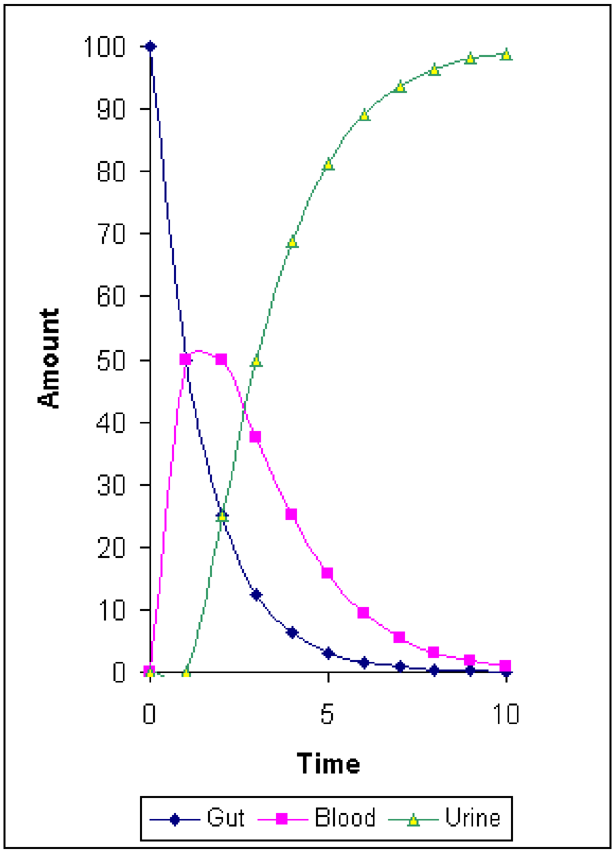

**FIGURE 2**. Simulated curves for absorption of drug from gut into blood and elimination into urine.

### Distribution to Tissues

The model is expanded by adding an extra column representing the tissues into which drug is distributed from the central compartment (blood). The equations in the cells for blood need to take into account the removal of drug from blood to tissues ($K_{12}$) and the redistribution of drug from tissue to blood ($K_{21}$); similarly the cells representing the tissue compartment need an equation for inflow from the blood and outflow to the blood. Fig. 3 shows a typical 60-min simulation for an oral drug dose and Fig. 4 shows the curves for an intravenous dose, using the same $K_e$, $K_{12}$, and $K_{21}$ values. For the oral dose, the initial value for the gut cell is 100; for the intravenous dose the initial value for the blood cell is 100.

By varying the values of $K_{12}$ and $K_{21}$, the effect of so-called deep and shallow compartments can be simulated. Further columns could be added to simulate deep and shallow compartments in the same model; the important point is that this is completely under the control of the modeller.

### Multiple Dosing

Using the spreadsheet approach, multiple dosing can be simulated simply by adding '+ 100' to the appropriate cell or cells within the spreadsheet (not copied to all cells but placed in the individual selected cells). However, this is a tedious procedure if the modeller wishes to see the effect of

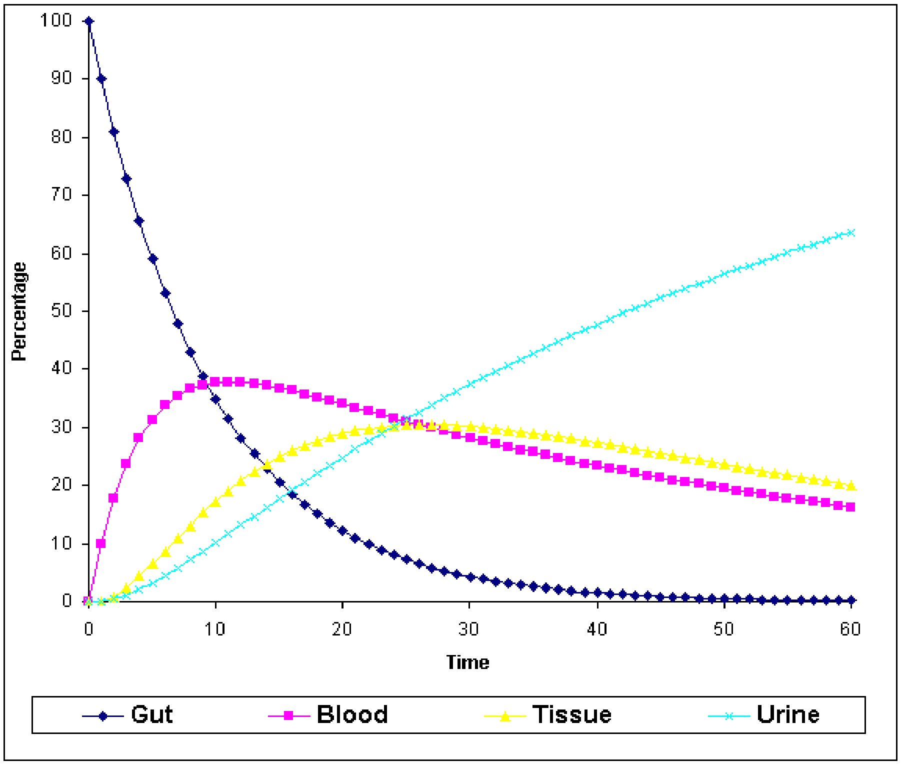

**FIGURE 3**. Typical simulation of absorption of drug from the gut, distribution into tissues, and elimination into urine.

changing doses or dose intervals. A more elegant solution is to use appropriate equations to enable variations to be keyed in once only. For example in the multiple dose spreadsheet included with this paper, the equation for the gut cell is of the form:

= I11 - I11*$D$4 + $K$4 * (H12/$K$5 = INT(H12/$K$5))

where I11 is the pre-existing value, $D$4 is the absorption coefficient ($K_a$), $K$4 is the repeated dose value, H12 is the current time, and $K$5 is the dosing interval. The term 'H12/$K$5=INT(H12/$K$5)' is only true when the current time is a multiple of the dosing interval (that is this expression returns a value of unity); otherwise the expression returns a value of zero and $K$4 is not added at this time point.

Fig. 5 shows a typical simulation for loading dose of 100 and a maintenance dose of 10 at 10-min intervals. As before, these are exaggerated values for the purposes of demonstration.

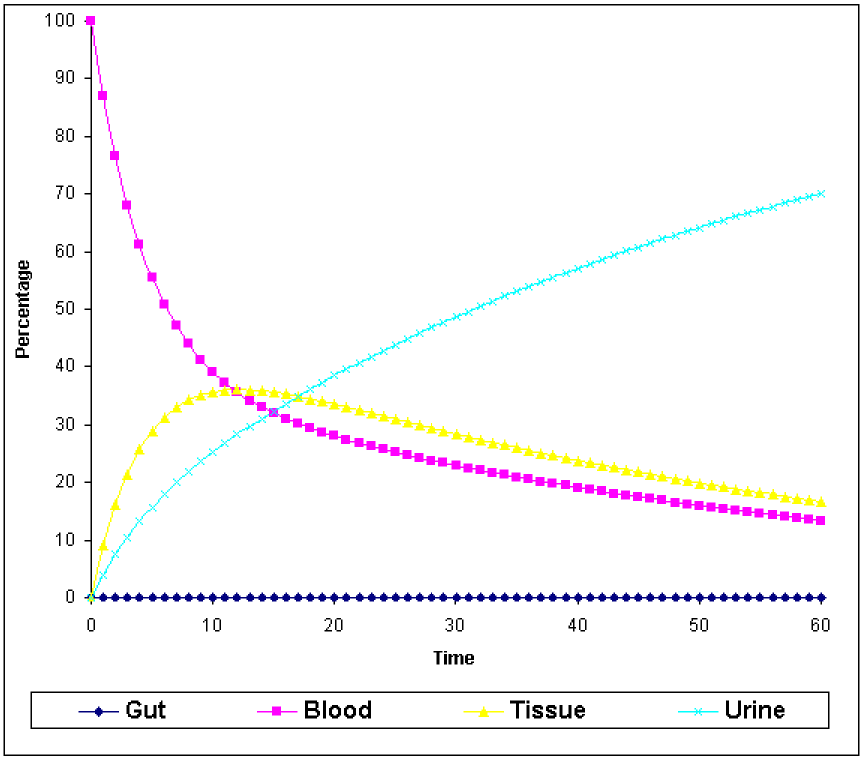

**FIGURE 4**. Typical simulation of distribution into tissues, and elimination into urine of an intravenously dosed drug.

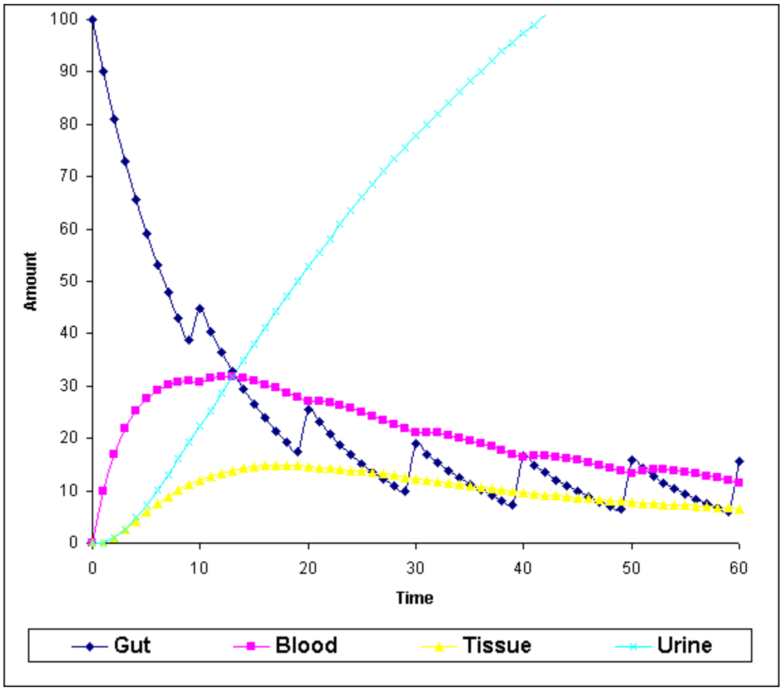

**FIGURE 5**. Typical simulation of multiple dose pharmacokinetics. The simulation shows a loading dose of 100 and a maintenance dose of 10 at 10-min intervals.

### Infusion

The same spreadsheet is used for simulating an infusion. Here it is the cells representing the blood compartment which are modified with the conditional terms:

= J11 + I11*$D$4 + K11*$D$7 - J11*$D$5 - J11*$D$6 + $K$6*(H12 < $K$7)

where J11 is the pre-existing value, I11 is the pre-existing value in the gut, $D$4 is the absorption coefficient, K11 is the pre-existing value in the tissues, $D$7 is the distribution coefficient from the tissues, $D$6 is the distribution coefficient to the tissues, $D$5 is the elimination coefficient, $K$6 is the infusion rate, H12 is the current time, and $K$7 is the infusion time. In this case $K$6 is only added to the blood value when H12 < $K$7, that is up to the end of the infusion period. Fig. 6 shows a simulation for an infusion rate of 10/min for 30 min.

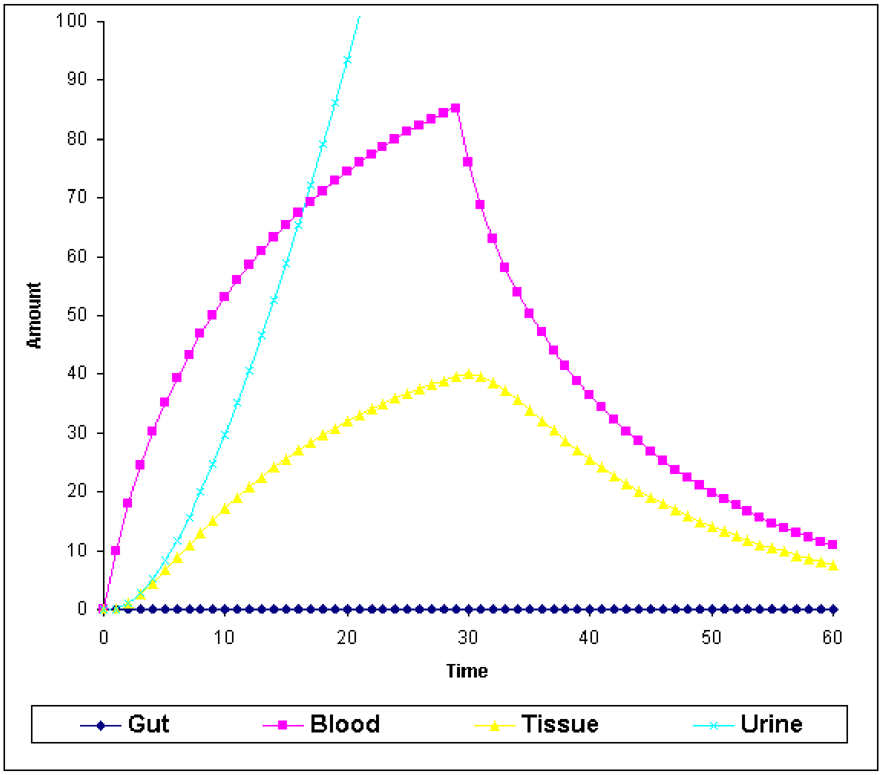

**FIGURE 6**. Typical simulation of intravenous pharmacokinetics. The simulation shows a continuous intravenous infusion of 10 units of dose per minute for a period of 30 min.

### Advanced Simulations

The spreadsheet can be expanded and developed to include any desired pharmacokinetic or metabolic feature. Some of these are illustrated in the spreadsheet attached to this paper and are described as follows.

### Gastrointestinal Absorption

Absorption does not take place at a single site following an oral dose of the drug. Rather the drug is moved down the gastrointestinal tract and may be absorbed at different rates depending on its location; thus absorption is a complex result of gastrointestinal transit and different rates of absorption into the portal vein. The demonstration spreadsheet assumes three compartments for the gastrointestinal tract (stomach, small intestine, large intestine), although the number of compartments can be increased as desired (e.g., duodenum, jejunum, ileum, segments of the colon, etc). A typical simulation is shown in Fig. 7.

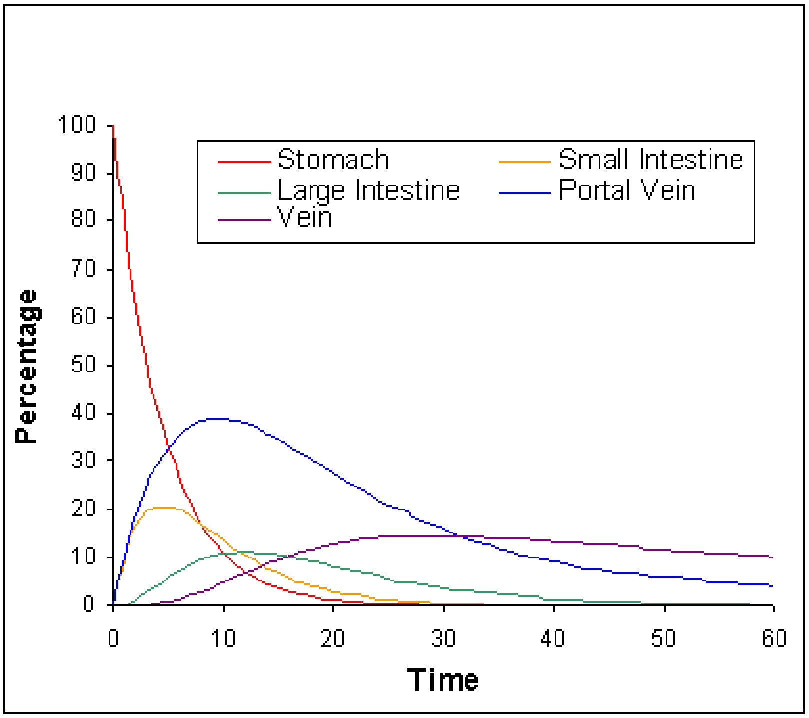

**FIGURE 7.** Simulation showing absorption of drug from different parts of the gastrointestinal tract.

### Exsorption

For these simulations, absorption is considered as passive diffusion through the gastrointestinal barrier. It follows therefore that if there is an excess concentration of drug in the portal vein then there is potential for some diffusion from the portal vein to the gastrointestinal tract. This can be readily simulated using the spreadsheet approach, so that even for an intravenous dose, some drug will appear in the gastrointestinal tract. Fig. 8 shows this when provision is made for exaggerated diffusion from the portal vein to the stomach.

### Gastric Emptying

A delay can be built in to any process by suitably modifying the spreadsheet. An example included in the demonstration is for gastric emptying. The appropriate formula is:

=F3 - F3 * ($C$4 + $C$18 * (E4>$C$23)) + I3*$C$6

where F3 is the pre-existing value of drug in the stomach, $C$4 is the absorption coefficient from stomach to portal vein, $C$18 is the first order process of transfer from stomach to small intestine, E4 is the current time, $C$23 is the delay in gastric emptying, I3 is the pre-existing value in the portal vein, and $C$6 is the coefficient of transfer from the portal vein to the stomach (to simulate the previous example on exsorption). An example where a 20-min delay before gastric emptying begins is shown in Fig. 9.

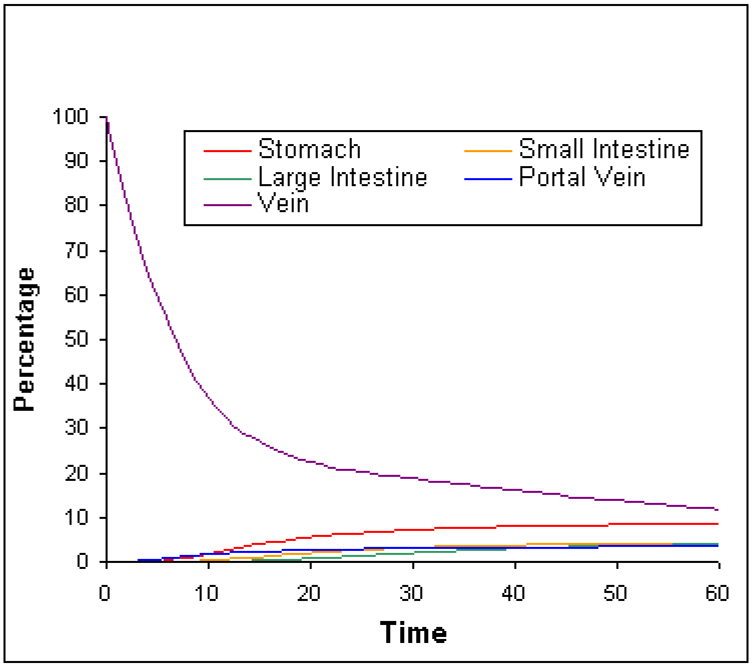

**FIGURE 8**. Simulation of an intravenous dose when provision is made for exsorption from the portal vein to the stomach. Drug then appears in the stomach and other parts of the gastrointestinal tract.

### Circulation and Distribution to Several Tissues

Although absorption of drug is to the portal vein, many classical pharmacokinetic texts assume direct absorption into the central measuring compartment, the systemic blood stream, or at least may consider the first-pass effect as part of the total absorption process. The spreadsheet example allows the modeller to include several compartments related to the circulation as well as distribution to the liver, the kidney, and other tissues as shown in Fig. 10. The rates of transfer from the blood vessels to the various tissues are related to relative blood flows and bring our model closer to a true physiological model.

### Enterohepatic Recirculation

The model detailed in Fig. 10 also shows provision for drug from the liver being transferred to the gall bladder. Some of this material is subsequently transferred into the small intestine and in this

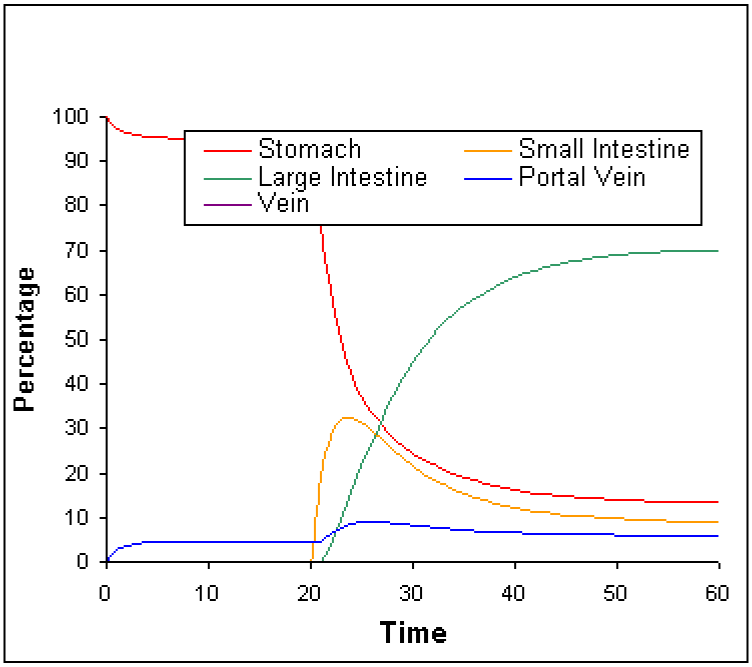

**FIGURE 9**. Simulation showing delayed absorption of drug from the gastrointestinal tract into the portal vein due to poor absorption from the stomach and delayed gastric emptying.

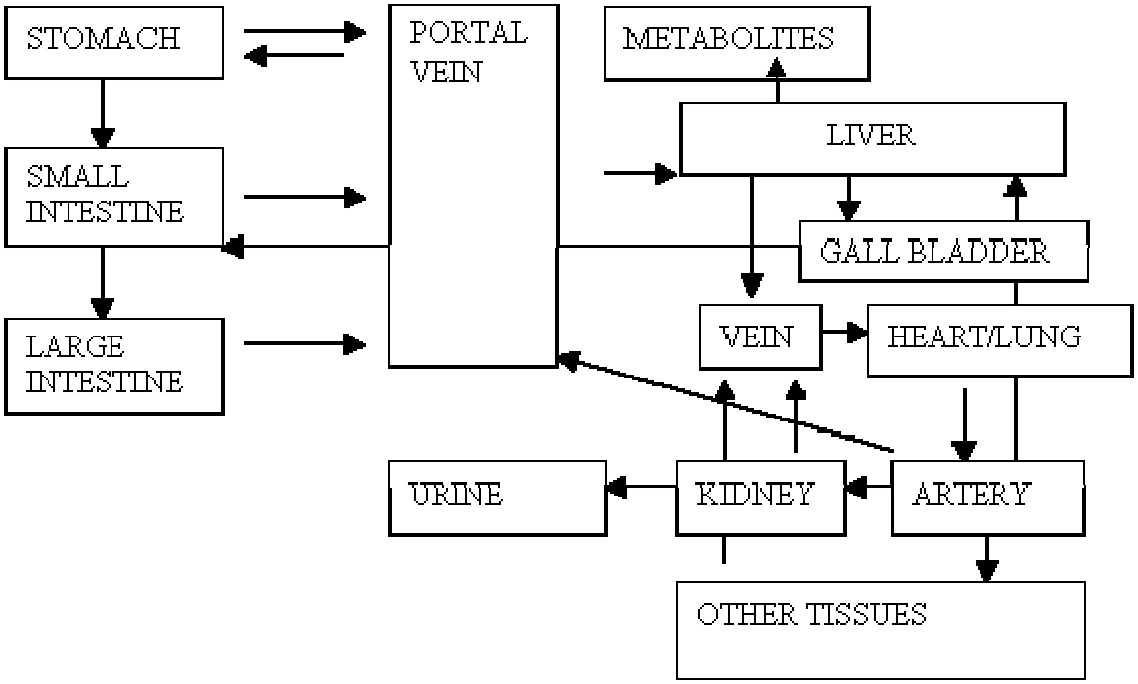

**FIGURE 10**. Schematic of a physiological model which can be readily simulated using the spreadsheet technique.

model this is arranged by entering the appropriate formulae in the cells for both the small intestine and the gall bladder. For example, the gall bladder contains the formula:

= R3 + J3*$C$10 - IF(E4=$C$22,R3/2,0)

where R3 is the pre-existing value in the gall bladder, J3 is the value in the liver, $C$10 is the transfer coefficient from liver to gall bladder, E4 is the current time, and $C$22 is the time at which the contents of the gall bladder are transferred; R3/2 ensures only half the content is transferred. Fig. 11 shows the outcome of this enterohepatic recirculation with the pronounced second hump in the plasma concentration time curve so often seen in typical pharmacokinetic experiments.

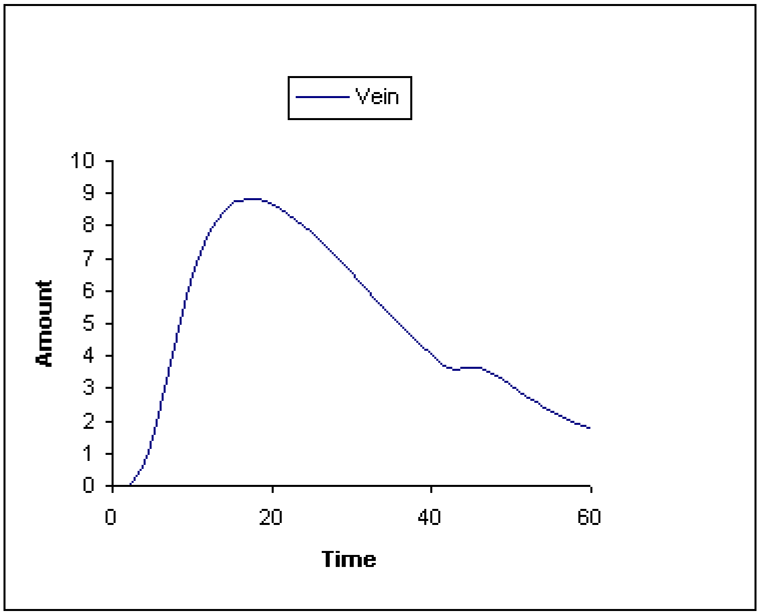

**FIGURE 11**. Simulation of blood concentration-time curve after an oral drug dose showing a second maximum due to enterohepatic recirculation.

### Metabolism

Not all transfers need to be first-order processes. Metabolism by such tissues as the liver is usually considered to proceed according to Michelis-Menten kinetics, which is dependent on $V_{max}$ and $K_m$ values. The formation of a metabolite is given by the formula:

= K3 + J3 * $C$42/(J3 + $C$41)

where K3 is the pre-existing amount of metabolite, J3 is the pre-existing amount of drug in the liver, $C$24 is the $V_{max}$ value for the enzyme/drug interaction and $C$41 is the $K_m$ value. In the demonstration spreadsheet, the formulation of two metabolites is modelled and their relative formation can be followed by varying the $V_{max}$ and $K_m$ values. A typical plot of the formation of two metabolites in the liver is shown in Fig. 12.

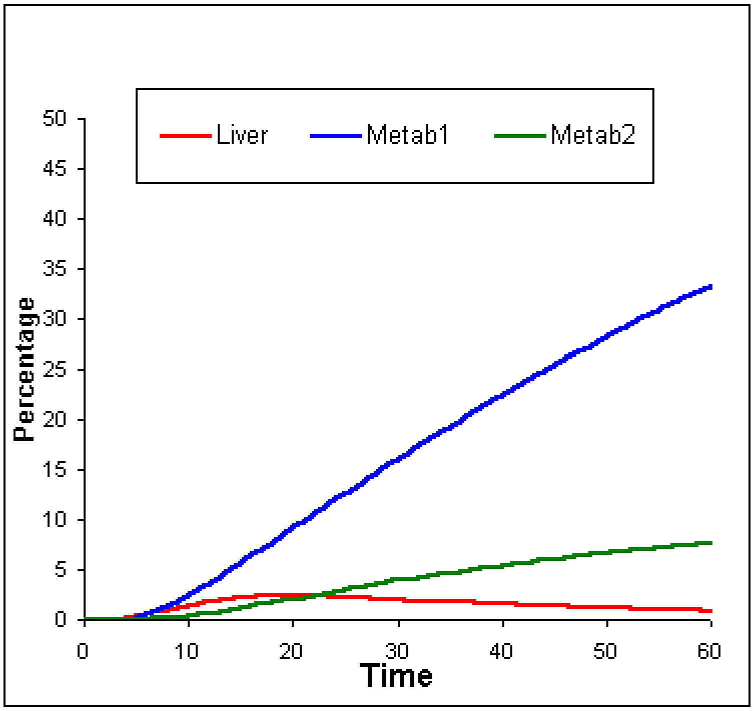

**FIGURE 12**. Formation of two metabolites in the liver. Metabolite 1 has a $K_m$ value of 1 and a $V_{max}$ value of 1; metabolite 2 has a $K_m$ value of 10 and a $V_{max}$ value of 1.

### Bioavailability

A measure of bioavailability is the ratio of the areas under the curves for the same oral and intravenous doses. In the spreadsheet example the relevant areas are computed simply by summing the values in the column representing the vein. The bioavailability (over the time scale shown) is then obtained after running the two simulations (oral and intravenous). In a typical example, under circumstances where less than 1% drug remained in the gastrointestinal tract after oral administration, the bioavailability, estimated from the model, was approximately 50%. When metabolism was removed (that is by setting $V_{max}$ values to zero), the bioavailability rose to greater than 70%. There is still a discrepancy however indicating that the classical definition of bioavailability is open to considerable reinterpretation.

### OTHER CONSIDERATIONS

The application and examples shown here have merely scratched the surface of this powerful technique. The model should be developed using both the full range of spreadsheet devices and further physiological, chemical, and biochemical parameters. For example, the time segments should be as short as possible but for long simulations this would mean a huge number of rows in the spreadsheet. However, macros can easily be constructed to transfer the values of the final row to the zero time point for as many times as one desires. The effect of lipophilicity or extent of ionisation can be included as modifying factors for the absorption coefficients to compare the fates of related molecules.

The predictive value of the spreadsheet approach depends on how far the modeller is prepared to go to develop a physiological model. Undoubtedly with true physiological, chemical, and biochemical values, the model can be very predictive. However, as the model becomes more complex it will run more slowly, and more conventional computer simulations will be more appropriate. Such an appropriate program is under development.

### CONCLUDING REMARKS

This paper describes how very complex pharmacokinetic models can be constructed using only standard spreadsheet packages and without recourse to complex calculus. In the main spreadsheet accompanying the paper a model is shown incorporating 14 compartments and 19 transfer or transformation processes. There is no limit to the number of compartments and processes that can be added; the whole process remains within the control of the modeller who will be continually stimulated to develop better and more accurate models.

The user of the present example may find the model may be unstable under certain circumstances, as the example is intended to demonstrate principles and not as a definitive program; spreadsheet devices to correct these instabilities would only add to the lengths of the formulae given and would obscure the messages intended in this exposition. Nevertheless, the reasons for any such instabilities should be carefully considered and the modeller will be able to improve the spreadsheet and hopefully his understanding of the underlining principles involved.

## Supplementary Material

All spreadsheets described are included in the attached file SpiresAdam.

